# Association of gait, balance, and handgrip strength with cognitive performance in hospitalized older adults: a retrospective analysis

**DOI:** 10.3389/fnagi.2026.1758286

**Published:** 2026-03-05

**Authors:** Deng-Peng Wen, Xiao Liu, Tian Gan, Jiang-Long Shi

**Affiliations:** 1 Department of Rehabilitation, Dazhou Dachuan District People’s Hospital (Dazhou Third People’s Hospital), Dazhou, Sichuan, China; 2 Department of Dushi Orthopedics, Sichuan Second Hospital of Traditional Chinese Medicine, Chengdu, China

**Keywords:** balance, cognitive impairment, gait speed, handgrip strength, Retrospective study, sensorimotor function

## Abstract

**Background:**

Sensorimotor impairments—such as reduced gait speed, diminished balance, and lower muscle strength—are common in older adults and have been suggested as early markers of cognitive decline. However, evidence from real-world hospital settings remains limited. This study investigated the associations between multiple sensorimotor functions and cognitive performance in hospitalized older adults.

**Methods:**

A retrospective cross-sectional study was conducted among 548 inpatients aged ≥ 60 years. Sensorimotor measures included gait speed, Timed Up and Go (TUG), handgrip strength, balance score, and activities of daily living (ADL). Cognitive performance was assessed using the Mini-Mental State Examination (MMSE). Correlations were analyzed using Pearson coefficients, followed by multivariable linear and logistic regression models adjusting for demographic, clinical, and laboratory covariates.

**Results:**

Only handgrip strength showed a significant positive correlation with MMSE score (*r* = 0.085, *P* = 0.046), whereas gait speed, TUG, balance score, and ADL were not significantly associated with cognitive performance. In multivariable linear regression, none of the sensorimotor measures independently predicted MMSE score after covariate adjustment. Education level was the strongest independent predictor of cognitive performance (β = 0.41, *P* < 0.001). In the fully adjusted logistic regression model, gait speed was significantly associated with cognitive impairment, whereas other sensorimotor indicators were not independently associated.

**Conclusion:**

In this real-world hospital cohort, sensorimotor measures were not independently associated with continuous MMSE performance after adjustment. However, gait speed was associated with cognitive impairment status in the fully adjusted logistic regression model, suggesting limited utility of routine sensorimotor assessments for cognitive screening during acute hospitalization. These findings should be interpreted cautiously given the retrospective, single-center design and potential measurement variability during hospitalization.

## Introduction

Aging is accompanied by progressive declines in both cognitive and motor systems, and the interplay between these domains has received increasing attention in geriatric research (Zapparoli et al., 2022). Cognitive impairment is highly prevalent among hospitalized older adults and is associated with adverse outcomes including prolonged hospital stay, increased risk of falls, functional decline, and higher mortality (Edison, 2020; McCollum and Karlawish, 2020; Han et al., 2023). Identifying simple, feasible markers that reflect cognitive status is therefore essential for early detection and clinical management.

Sensorimotor function—including gait speed, balance, handgrip strength, and mobility performance—has been proposed as a potential window into brain health (Spence et al., 2024; Smith et al., 2018). Prior studies have shown that slower gait, poorer balance, and reduced muscle strength may reflect disruptions in neural circuits that integrate motor and cognitive processes (Lugade et al., 2014; Wu et al., 2023). Handgrip strength, in particular, has been linked to executive function, global cognition, and frailty (Caetano et al., 2017). However, much of the existing evidence is derived from community-based cohorts, rehabilitation settings, or longitudinal population studies. Whether these sensorimotor markers retain their predictive value in real-world hospital environments, where patients often have multimorbidity and acute physiological stress, remains unclear.

Hospitalized older adults represent a unique population with increasing research attention. Functional assessments are conducted routinely in many clinical settings, yet the specific associations between routinely assessed sensorimotor measures and concurrent cognitive performance during acute or subacute hospitalization remain insufficiently characterized, particularly in real-world inpatient settings. Furthermore, findings from previous studies are inconsistent, and it is uncertain whether sensorimotor traits independently predict cognitive status after accounting for demographic factors, comorbidities, and laboratory indicators of overall health. Clarifying these associations may help clinicians identify cognitive vulnerability using measures that are simple, non-invasive, and already embedded in routine practice.

Therefore, the present study aimed to examine the associations between multiple sensorimotor functions—gait speed, Timed Up and Go (TUG), handgrip strength, balance performance, and activities of daily living (ADL)—and cognitive status among hospitalized older adults. Using a retrospective cross-sectional design based on real-world data from a regional hospital in China, we evaluated both continuous cognitive performance (the Mini-Mental State Examination, MMSE) and cognitive impairment as outcomes. We hypothesized that certain sensorimotor measures, particularly handgrip strength and gait performance, might demonstrate measurable associations with cognition even in a hospitalized cohort.

## Materials and methods

### Study design

This study was conducted as a retrospective cross-sectional analysis using routinely collected inpatient data. The primary aim was to examine the associations between multiple sensorimotor functions and cognitive performance in hospitalized older adults. All available clinical, functional, and laboratory information recorded during routine care was extracted from the hospital electronic medical record system. No interventions or prospective measurements were performed, and no data outside routine clinical practice were collected.

This study was reported in accordance with the STROBE guideline for cross-sectional studies and the RECORD guideline for studies using routinely collected health data. The completed checklists are provided as Supplementary material.

### Setting and participants

All data were obtained from the electronic medical record system of Dachuan District People’s Hospital of Dazhou City, Sichuan Province, China. Patients admitted between January 2023 and December 2025 were screened for eligibility. Older inpatients aged 60 years or above who were admitted during the study period and who had completed cognitive assessment were eligible for inclusion. Patients were screened based on the availability of MMSE results and at least one sensorimotor measure. Individuals were excluded if they lacked essential demographic, clinical, or functional information or if key assessments were incomplete. After applying these criteria, a total of 548 older adults were included in the final analysis. Participants were admitted for a range of medical or functional conditions; cognitive impairment was not the primary reason for hospitalization.

All patients provided general authorization for the use of anonymized clinical data for research purposes at hospital admission.

### Sensorimotor measures

Sensorimotor function was assessed using routinely conducted clinical evaluations. Gait speed was measured using a standardized 4-m walking test, in which participants were instructed to walk at their usual pace. Gait speed (m/s) was calculated as the distance divided by the time required to complete the walk. The TUG test recorded the time required for the participant to rise from a chair, walk three meters, turn around, return, and sit down. Handgrip strength was measured with a handheld dynamometer, and the maximal value from either hand was used for analysis. Balance ability was evaluated using a standardized clinical balance test scored on a 0–4 scale, with higher scores indicating better postural stability. Functional status in daily life was assessed using the activities of daily living (ADL) scale, ranging from 0 to 6, where higher scores reflect greater independence.

### Cognitive assessment

Cognitive performance was measured using the MMSE, administered by trained clinical staff as part of routine evaluation of older inpatients. The MMSE provides a global cognitive score ranging from 0 to 30. Cognitive impairment was defined as an MMSE score < 24, consistent with widely adopted clinical and research standards for older adult populations.

### Covariates

Demographic variables including age, sex, and years of education were extracted from the medical records. Body mass index (BMI) and major comorbidities, including hypertension and diabetes, were obtained from physician documentation and laboratory records. Laboratory variables included C-reactive protein (CRP), albumin (ALB), hemoglobin (Hb), and estimated glomerular filtration rate (eGFR). These covariates were selected based on their clinical relevance and previously reported associations with sensorimotor performance or cognitive function.

### Statistical analysis

All statistical analyses were performed using R and Python. Continuous variables were described as means ± standard deviations, and categorical variables were summarized as percentages. Group comparisons between cognitively impaired and non-impaired participants were conducted using independent-sample *t*-tests for continuous variables and chi-square tests for categorical variables. Pearson correlation coefficients were calculated to examine associations between MMSE scores and each sensorimotor measure. To assess independent associations, multivariable linear regression models were constructed with MMSE score as the dependent variable, adjusting for demographic, clinical, and laboratory covariates. In addition, multivariable logistic regression analyses were performed to evaluate predictors of cognitive impairment (MMSE < 24). Multivariable linear and logistic regression models were considered the primary inferential analyses, while correlation analyses were conducted for exploratory and descriptive purposes only. Results from the logistic models were presented as odds ratios (ORs) with 95% confidence intervals. Statistical significance was defined as a two-sided *P* < 0.05.

### Ethical considerations

This study was conducted in accordance with the principles of the Declaration of Helsinki. Because all data were retrospectively extracted from existing clinical records and fully anonymized prior to analysis, the study received an ethics exemption from the Ethics Committee of Dachuan District People’s Hospital of Dazhou City, and the requirement for informed consent was waived.

## Results

### Baseline characteristics

The study included 548 hospitalized older adults. Table 1 presents the baseline characteristics stratified by cognitive status. No significant differences were observed between participants with and without cognitive impairment across demographic variables (age, sex, education, BMI) or clinical factors (hypertension, diabetes, CRP, albumin, hemoglobin, and eGFR). Similarly, all sensorimotor measures—including gait speed, TUG performance, grip strength, balance score, and ADL—were comparable between groups (all *P* > 0.05).

**TABLE 1:** Baseline characteristics of hospitalized older adults.

Variable	Non-impaired	Impaired	*P*-value
Age, years	72.11 ± 7.16	72.50 ± 6.48	0.522
Sex, male (%)	47.10%	47.70%	0.897
Education, years	7.97 ± 3.29	8.47 ± 2.83	0.068
BMI	23.70 ± 3.31	23.32 ± 3.07	0.278
Gait speed (m/s)	0.739 ± 0.178	0.735 ± 0.179	0.829
TUG (sec)	14.30 ± 3.84	14.41 ± 4.12	0.728
Grip strength (kg)	22.14 ± 6.36	21.95 ± 6.22	0.715
Balance score (0–4)	2.13 ± 0.91	2.02 ± 0.87	0.228
ADL (0–6)	4.07 ± 1.30	4.18 ± 1.29	0.327
Hypertension (%)	55.80%	57.10%	0.765
Diabetes (%)	22.00%	22.80%	0.823
CRP (mg/L)	5.72 ± 5.60	5.51 ± 5.23	0.693
Albumin (g/L)	37.92 ± 4.05	37.68 ± 4.01	0.468
Hemoglobin (g/L)	124.25 ± 16.59	124.15 ± 15.96	0.344
eGFR	70.30 ± 21.06	69.82 ± 20.40	0.795

### Correlations between sensorimotor measures and cognitive performance

As shown in Table 2, grip strength demonstrated a very weak unadjusted correlation, which reached nominal statistical significance but explained only a minimal proportion of variance in MMSE (*r* = 0.085, *P* = 0.046) scores. No significant correlations were found between MMSE and gait speed (*P* = 0.165), TUG (*P* = 0.264), balance score (*P* = 0.454), or ADL (*P* = 0.753). These results suggest that among the evaluated sensorimotor domains, only handgrip strength showed a measurable relationship with overall cognitive performance.

**TABLE 2:** Pearson correlations between sensorimotor function and MMSE.

Sensorimotor measure	*r*	*P*-value
Gait speed (m/s)	0.059	0.165
TUG (sec)	0.048	0.264
Grip strength (kg)	0.085	0.046
Balance score	0.032	0.454
ADL	−0.013	0.753

**FIGURE 1:**
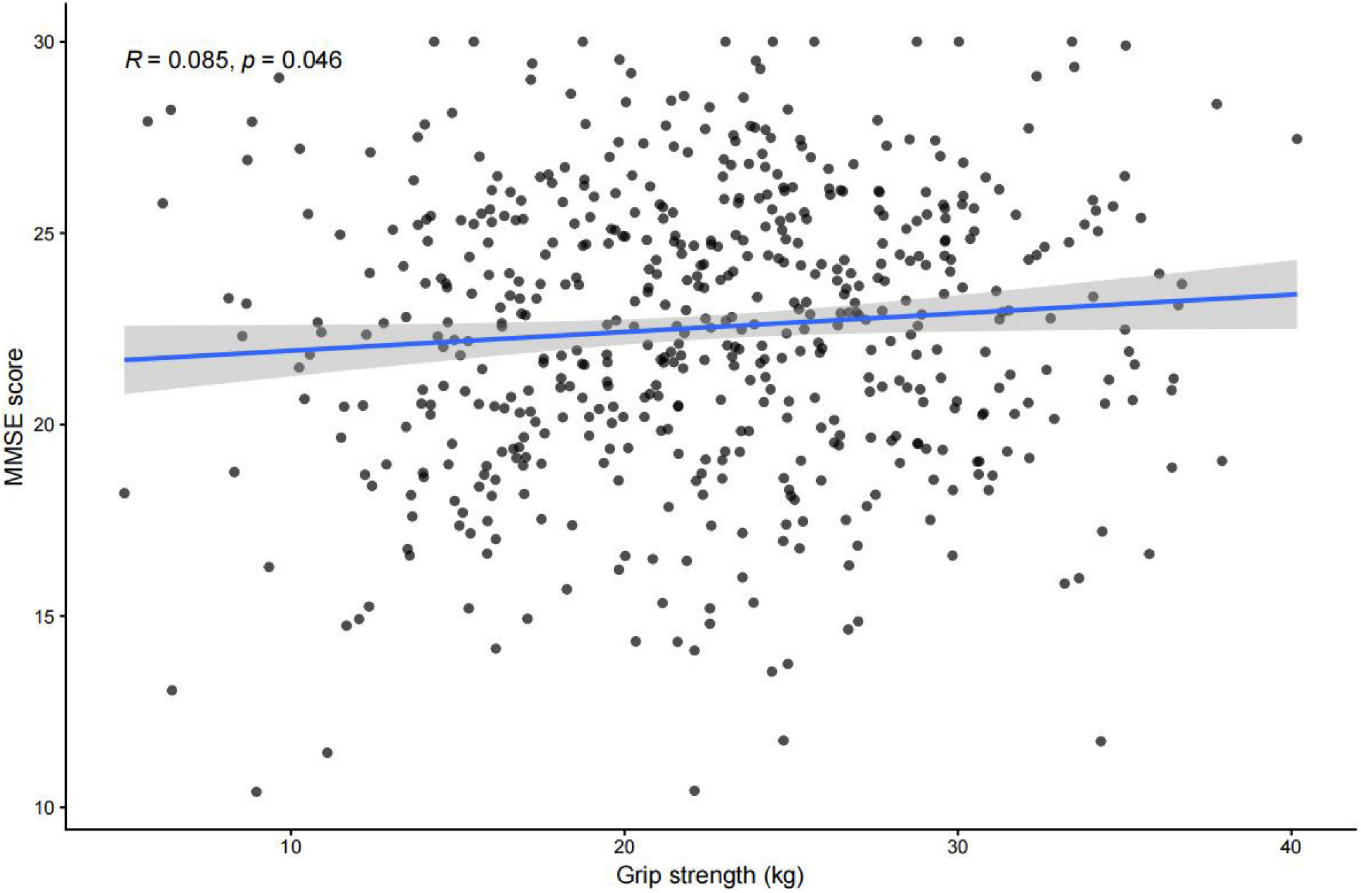
Scatter plot illustrating the association between handgrip strength and MMSE score in hospitalized older adults. Each point represents one participant, and the blue line indicates the fitted linear regression with 95% confidence interval (gray shading). A weak but statistically significant positive correlation was observed (*r* = 0.085, *p* = 0.046).

As illustrated in Figure 1, a weak but statistically significant positive association was observed between grip strength and MMSE score (*r* = 0.085, *p* = 0.046). The scatter plot with fitted regression line shows a slight upward trend, indicating that higher handgrip strength was modestly associated with better cognitive performance. No other sensorimotor variable demonstrated a significant correlation.

### Multivariable linear regression predicting MMSE

TO Assess Independent associations between sensorimotor function and cognitive performance, a multivariable linear regression model was constructed (Table 3). After adjustment for age, sex, education, BMI, comorbidities, and laboratory markers, none of the sensorimotor indicators (gait speed, TUG, grip strength, balance score, or ADL) were independently associated with MMSE scores (all *P* > 0.05). Education level emerged as the strongest independent predictor of cognitive performance (β = 0.41, 95% CI: 0.29–0.53, *P* < 0.001).

**TABLE 3:** Multivariable linear regression predicting MMSE.

Variable	β coefficient	95% CI	*P*-value
Gait speed	−0.98	−3.42 to 1.46	0.425
TUG	0.02	−0.09 to 0.13	0.734
Grip strength	0.03	−0.03 to 0.09	0.325
Balance score	0.1	−0.24 to 0.44	0.563
ADL	−0.07	−0.47 to 0.33	0.736
Age	−0.01	−0.08 to 0.05	0.692
Sex (male)	0.43	−0.29 to 1.16	0.238
Education	0.41	0.29 to 0.53	< <0.001
BMI	0.02	−0.07 to 0.11	0.676
Hypertension	−0.13	−0.89 to 0.63	0.739
Diabetes	−0.08	−1.00 to 0.84	0.864
CRP	−0.01	−0.04 to 0.03	0.828
Albumin	0.03	−0.04 to 0.11	0.378
Hemoglobin	0.002	−0.02 to 0.02	0.898
eGFR	−0.01	−0.01 to 0.01	0.871

### Logistic regression predicting cognitive impairment

**FIGURE 2:**
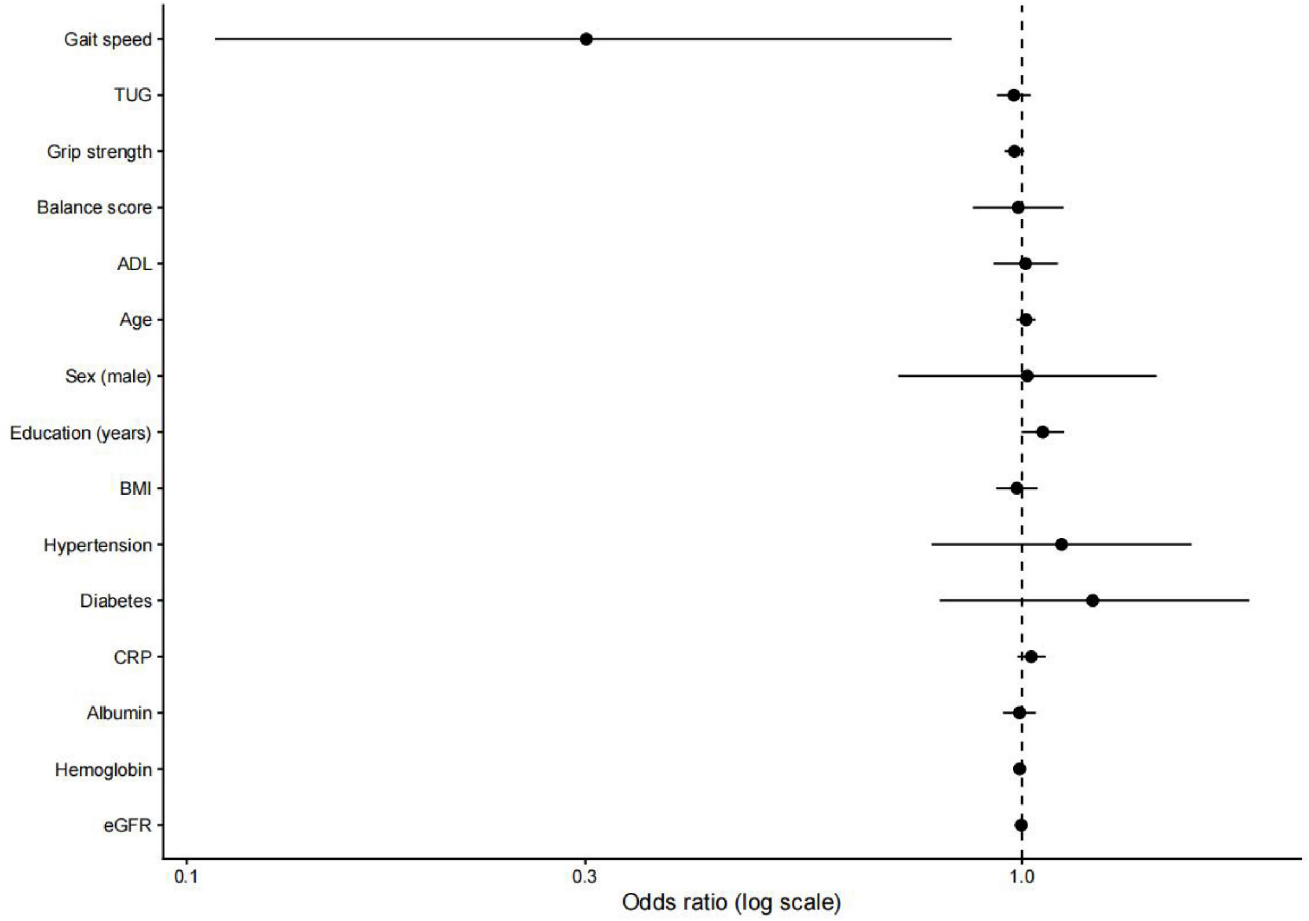
Logistic regression analysis of predictors of cognitive impairment. Summary of key findings.

In the fully adjusted logistic regression model (Figure 2), gait speed was significantly associated with cognitive impairment, with slower gait speed associated with higher odds of impairment. In contrast, TUG performance, handgrip strength, balance score, and ADL were not independently associated with cognitive impairment after adjustment for demographic, clinical, and laboratory variables. Higher educational attainment was also significantly associated with cognitive impairment. No other covariates showed statistically significant associations.

### Summary of key findings

Across all analyses, sensorimotor performance was not independently associated with continuous cognitive performance as measured by MMSE. In contrast, gait speed was associated with cognitive impairment status in the fully adjusted logistic regression model, whereas other sensorimotor indicators were not independently associated.

## Discussion

The present study examined the associations between multiple sensorimotor functions and cognitive status in a real-world cohort of hospitalized older adults. Using routinely collected clinical assessments, we found that although handgrip strength showed a weak unadjusted correlation with global cognitive performance, none of the sensorimotor measures were independently associated with MMSE score, whereas gait speed was associated with cognitive impairment status in the fully adjusted logistic regression model. Education level emerged as the most consistent predictor of cognitive function across all models. These findings suggest that sensorimotor performance may have limited predictive value for cognitive status in acutely or subacutely hospitalized older adults, contrasting with results reported in community-based cohorts.

Our results differ from many population studies in which gait speed, balance impairment, and muscle weakness have been linked to cognitive decline and dementia risk (Meng et al., 2019; Makizako et al., 2011; Evidente and Gwinn, 1997). Longitudinal cohorts such as the Health ABC Study, the English Longitudinal Study of Aging, and the InCHIANTI Study consistently demonstrate that slower gait and reduced strength precede and predict future cognitive decline (Martin et al., 2013; Sloane and Warshaw, 2022). Similarly, previous meta-analyses have identified handgrip strength as a robust correlate of cognitive aging (Rijk et al., 2016). However, these associations may not generalize to inpatient populations. Hospitalized older adults often present with acute illness, functional fluctuations, inflammation, and multimorbidity, which may obscure or attenuate the relationship between chronic sensorimotor decline and cognitive status (Loyd et al., 2020; Ellis et al., 2017; McDonagh et al., 2025; Thomas et al., 2023). In addition, cognitive performance measured during hospitalization may reflect both baseline cognition and acute physiological stressors, reducing the strength of associations observed in stable community settings (Glans et al., 2023; Jonsson et al., 2017; Nouvenne et al., 2022).

Another possible explanation is that sensorimotor function in hospitalized patients may be influenced by short-term factors such as fatigue, pain, limited mobility during admission, or acute deconditioning (Lafuente-Lafuente et al., 2025; Naughton et al., 2011). These factors may introduce variability unrelated to neurocognitive processes. Furthermore, routine clinical assessments—while practical and widely used—may lack the sensitivity of research-grade gait analysis or strength measurement tools. Together, these factors may reduce the signal-to-noise ratio and weaken observable associations between sensorimotor and cognitive domains in the inpatient environment.

The finding that education was the strongest predictor of cognitive performance is consistent with cognitive reserve theory, which posits that individuals with higher educational attainment may maintain better cognitive functioning despite age-related or pathological changes (Berkes et al., 2020; Stern, 2006; van Loenhoud et al., 2019). This effect has been repeatedly demonstrated across diverse populations and settings, including hospitalized older adults. Education may therefore represent a more stable and robust indicator of cognitive vulnerability in clinical settings than sensorimotor measures influenced by acute health fluctuations.

Despite the lack of strong associations between sensorimotor function and cognition in the present cohort, our study has important clinical implications. First, grip strength demonstrated a modest correlation with MMSE, suggesting that it may still serve as a quick screening indicator for identifying individuals who may benefit from further cognitive evaluation. Second, the findings underscore the importance of considering contextual factors—such as acute illness and hospitalization—when interpreting sensorimotor assessments in older adults. Finally, the results highlight the need for longitudinal follow-up to determine whether sensorimotor performance during hospitalization predicts long-term cognitive outcomes, such as post-hospitalization cognitive decline or functional recovery.

This study has several limitations that should be considered when interpreting the certainty of the findings. First, the retrospective design relied on routinely collected clinical data, which may be affected by missingness and measurement variability. Such non-differential measurement error—particularly for bedside functional assessments—would be expected to attenuate true associations toward the null, thereby reducing confidence in the absence of independent relationships. Second, sensorimotor assessments were performed during acute or subacute hospitalization; short-term factors (e.g., fatigue, pain, acute deconditioning, mobility restrictions, or delirium risk) may have influenced performance and increased within-person variability, again potentially diluting associations with cognitive outcomes. Third, the MMSE has limited sensitivity for mild cognitive impairment and may exhibit ceiling effects, which could compress score variability and further bias effect estimates toward null findings in linear models. Fourth, although we adjusted for major demographic, clinical, and laboratory covariates, residual confounding remains possible—particularly from medication exposure, neuropsychiatric symptoms, and illness severity—which may either obscure or inflate associations and therefore limits certainty regarding independence. Finally, the single-center setting may constrain external validity; thus, our results may be most applicable to similar inpatient populations and should be confirmed in prospective multicenter studies with standardized assessment timing and more granular confounder measurement.

This retrospective analysis of hospitalized older adults found that sensorimotor measures—including gait speed, TUG, grip strength, balance, and ADL—were not independently associated with cognitive performance after adjusting for key clinical and demographic variables. Education level remained the strongest predictor of cognitive status. These findings suggest that the utility of sensorimotor assessments for cognitive screening may be limited in inpatient populations. Future prospective and longitudinal studies are warranted to clarify the temporal relationships between motor and cognitive decline and to identify more stable predictors of cognitive vulnerability during hospitalization.

## Conclusion

In this retrospective analysis of hospitalized older adults, sensorimotor measures were not independently associated with global cognitive performance as measured by MMSE. However, gait speed was associated with cognitive impairment status in the fully adjusted logistic regression model. Education level emerged as the most consistent predictor of cognitive status, underscoring the importance of cognitive reserve in this setting. These findings suggest that sensorimotor assessments obtained during hospitalization may have limited value for identifying cognitive vulnerability, likely due to the influence of acute illness and functional fluctuations. Future longitudinal studies are needed to determine whether sensorimotor performance measured during hospitalization has prognostic value for post-discharge cognitive trajectories and functional recovery.

## Data Availability

The original contributions presented in this study are included in this article/Supplementary material, further inquiries can be directed to this corresponding author.
